# Neutron diffraction analysis of stress and strain partitioning in a two-phase microstructure with parallel-aligned phases

**DOI:** 10.1038/s41598-020-70299-1

**Published:** 2020-08-11

**Authors:** Qiuliang Huang, Ran Shi, Ondrej Muránsky, Hossein Beladi, Saurabh Kabra, Christian Schimpf, Olena Volkova, Horst Biermann, Javad Mola

**Affiliations:** 1 Institute of Iron and Steel Technology, Technische Universität Bergakademie Freiberg, Leipziger Str. 34, 09599 Freiberg, Germany; 2 Material Design and Structural Integrity Lab, Faculty of Engineering and Computer Sciences, Osnabrück University of Applied Sciences, Albrecht St. 30, 49076 Osnabrück, Germany; 3 Australian Nuclear Science and Technology Organisation (ANSTO), Sydney, NSW 2234 Australia; 4 Institute for Frontier Materials, Deakin University, Geelong, VIC 3216 Australia; 5 Spallation Neutron Source, The Rutherford Appleton Laboratory, Oxfordshire, UK; 6 Institute of Materials Science, Technische Universität Bergakademie Freiberg, Gustav-Zeuner-Straße 5, 09599 Freiberg, Germany; 7 Institute of Materials Engineering, Technische Universität Bergakademie Freiberg, Gustav-Zeuner-Straße 5, 09599 Freiberg, Germany; 8 Present Address: Institute of Energy Process Engineering and Chemical Engineering, Fuchsmühlenweg 9, 09599 Freiberg, Germany

**Keywords:** Mechanical properties, Metals and alloys

## Abstract

By time-of-flight (TOF) neutron diffraction experiments, the influence of segregation-induced microstructure bands of austenite (***γ***) and martensite (***α′*** ) phases on the partitioning of stress and strain between these phases was investigated. Initially, tensile specimens of a Co-added stainless steel were heat treated by quenching and partitioning (Q&P) processing. Tensile specimens were subsequently loaded at 350 °C parallel to the length of the bands within the apparent elastic limit of the phase mixture. Lattice parameters in both axial and transverse directions were simultaneously measured for both phases. The observation of a lattice expansion for the ***γ*** phase in the transverse direction indicated a constraint on the free transverse straining of ***γ*** arising from the banded microstructure. The lateral contraction of ***α′*** imposed an interphase tensile microstress in the transverse direction of the ***γ*** phase. The multiaxial stress state developed in the ***γ*** phase resulted in a large deviation from the level of plastic strain expected for uniaxial loading of single phase* γ*. Since segregation-induced banded microstructures commonly occur in many engineering alloys, the analysis of stress and strain partitioning with the present Q&P steel can be used to interpret the observations made for further engineering alloys with two-phase microstructures.

## Introduction

Ever since its introduction in 2003^1^, the quenching and partitioning (Q&P) processing has attracted increasing interest for developing advanced high strength steels^2–6^. By virtue of the presence of retained austenite (RA) with sufficient mechanical stability, Q&P steels can exhibit an enhanced ductility-strength combination compared to quenched and tempered steels^7,8^. In the course of processing, austenite (***γ***) is first partially transformed to martensite (***α′*** ) by quenching to temperatures below martensite start temperature (M_s_). Upon subsequent reheating, the supersaturated carbon (C) in the martensite diffuses into austenite and thereby stabilizes austenite. This process is referred to as “partitioning”.

The enrichment of C in the austenite phase of Q&P steels during partitioning is associated with a lattice expansion^9^. Interpreting the extent of partitioning based on the increase in the lattice parameter might become difficult owing to the overlap of several competing phenomena during the partitioning step. Challenges include the simultaneous occurrence of bainite transformation and the decomposition of RA, which can also contribute to C enrichment into the remaining austenite and hence overlap with the C partitioning from martensite into austenite^10^. The efficiency of Cr in suppressing the bainitic reaction implies that stainless steels such as the one used in present work can be processed via Q&P processing without concerns about the bainite formation in the partitioning step^8^. Furthermore, due to the high Cr content of stainless steels, the decomposition of RA in stainless steels can only occur subsequent to its destabilization by the formation of Cr-rich carbides^11^. During dilatometry heating of Fe–13Cr–0.47C and Fe–13Cr–2Si–0.47C (concentrations in wt% throughout the text) steels at a rate of 5 K/min, an increase in the apparent coefficients of thermal expansion (CTE) caused by the decomposition of RA to a mixture of ferrite and carbides was detected at temperatures higher than 540 °C^12^.

Upon quenching, martensitic transformation is accompanied by a shape change consisting of a shear strain of approximately 0.22 and a dilatational strain of 0.03 normal to the habit plane^13,14^. In steels consisting of compositional bands created by hot rolling or hot extrusion, the length changes during martensitic transformation have been observed to be anisotropic along the longitudinal and transverse directions^15,16^. These observations were attributed to the interdendritic segregation of alloying elements during solidification and the parallel alignment of the segregated regions during hot processing, eventually leading to the formation of bands with different martensite start and austenite reversion temperatures. Anisotropic dimensional changes were also observed during cyclic annealing of a forged duplex stainless steel rod with a preferential alignment of phases^17^. The results showed that the longitudinal specimen expanded with each cycle, whereas the transverse specimen contracted. These were interpreted in view of the dissimilar CTE of ***γ*** and ferrite (***α***) that coexisted in the banded structure. Anisotropic length changes during the martensitic transformation of austenite in a ferritic stainless steel consisting of ***γ + α*** at high temperatures have been attributed to the planar banded arrangement of phases caused by the prior hot rolling^18^. In the latter case, ferrite acted as a constraint against the in-rolling-plane strain (strain parallel to the bands) during the transformation, causing exaggerated dimensional changes perpendicular to the bands, namely in the normal direction.

In addition, the deformation behavior is far from being homogeneous in alloys consisting of several phases characterized by different flow stress levels, e.g. the ***γ*** and ***α*** phases in duplex stainless steels^19^. The partitioning of stress and strain in two-phase composite materials is rather complex as it may be influenced by factors such as the stress–strain behavior of each single phase, spatial distribution of phases, and their volume fractions^20,21^. In particular, a large difference exists whether it is the harder or the softer phase that constitutes the matrix^22^. Since the microstructure of most advanced steels consists of multiple phases with different mechanical properties and a non-homogenous distribution of phases, it is important to identify the influence of such inhomogeneities on the ability of phases to carry stress and strain and to relate this with the mechanical properties.

The evolution of lattice parameter in constituent phases of multiphase alloys can be experimentally determined by using diffraction techniques^23^. The selective nature of diffraction techniques enables to separate the elastic response of different phases during deformation and separate the elastic portion of the total deformation from the plastic part. Mary et al.^24^ quantified the residual stresses in ***α*** and ***γ*** phases present in the banded microstructure of a hot-rolled duplex stainless steel using X-ray diffraction (XRD). The time-of-flight (TOF) neutron diffraction technique used in the current study is a well-established tool for studying micromechanical behavior of materials^25,26^. Information from the exposed volume can be simultaneously collected by two detectors placed perpendicularly to each other, enabling to calculate the stress and strain in two perpendicular directions.

The present work analyses the partitioning of stress and strain between martensite and austenite phases in a Q&P stainless steel by means of in-situ TOF neutron diffraction. The results highlight the importance of compositional banding, an inevitable characteristic of many engineering alloys, on the partitioning of stress and strain and yielding of the coexisting phases.

## Methods

The studied steel, a Co-containing stainless steel with the chemical composition given in Table 1, was produced in a vacuum induction melting and casting facility. After homogenization at 1,150 °C for 3 h, the cast ingot was forged and caliber-rolled into a bar with a diameter of 12 mm. Tensile specimens parallel to the rolling direction (RD) were machined in accordance with the DIN 50125 standard and had a gauge diameter of 6 mm and a parallel length of 36 mm.

**Table 1:** Chemical composition of the investigated stainless steel (in wt%).

Steel	C	Cr	Co	Si	Mn	N	Fe + others
CrCo	0.46	12.6	3.07	0.34	0.31	0.009	Bal.

As the quenching and partitioning steps were performed separately at different locations, room temperature (RT) was selected as quench temperature to facilitate the transportation of the specimens. Prior to quenching, the specimens were solution annealed at 1,250 °C for 10 min in a sealed quartz tube evacuated to 2.5 × 10^–3^ mbar before sealing. After breaking the tube, the specimens were quenched in an oil bath at 60 °C followed by air cooling to RT.

**Figure 1:**
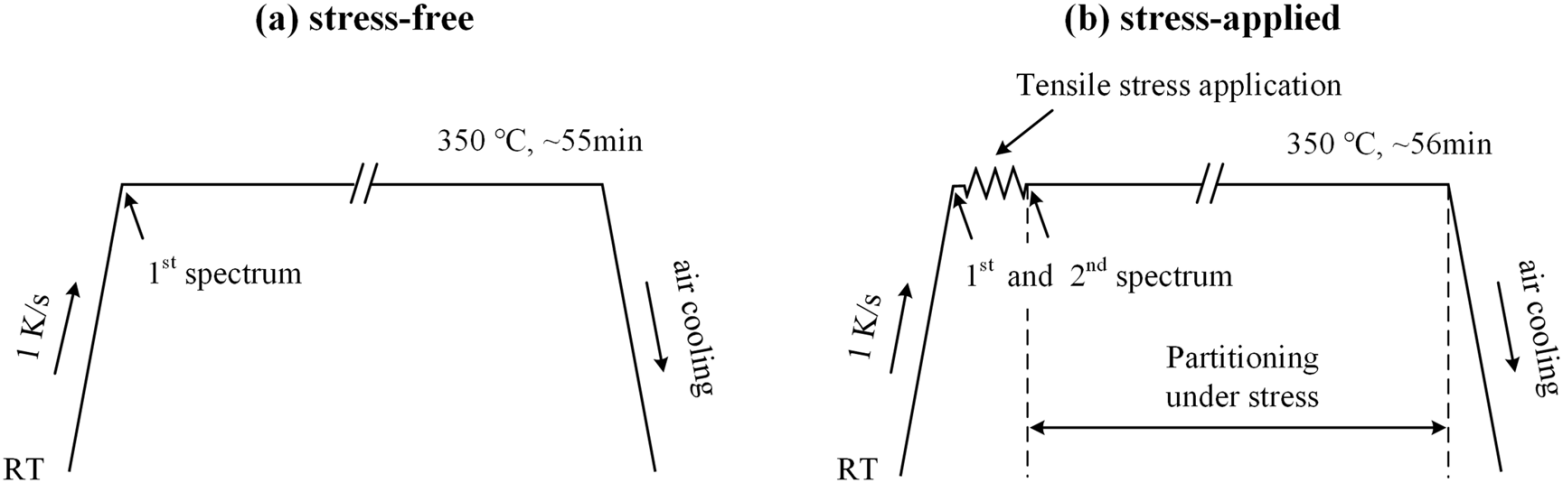
Schematic diagrams of the reheating schemes for (**a**) stress-free and (**b**) stress-applied measurements.

Diffraction measurements were carried out within the ENGIN-X diffractometer equipped with a heating stage and a stress rig in the ISIS spallation neutron source, the Rutherford Appleton Laboratory, UK. The diffractometer enabled in-situ TOF neutron diffraction measurements. Diffraction measurements were done once the temperature reached 350 °C. Temperature was measured by a thermocouple attached to the gauge section of the tensile specimen. The choice of a high temperature, rather than RT, for the in-situ neutron diffraction measurements was intended to exclude the deformation-induced transformation of austenite. The routines for stress-free and stress-applied measurements are schematically shown in Fig. 1. In both experiments, the specimens were first reheated at about 1 K/s to a partitioning temperature of 350 °C in a radiant air furnace followed by the collection of the first diffraction pattern. During the stress-free experiment, further diffraction patterns were continuously recorded. The counting time for each spectrum was 2.5 min. Measurements were terminated after the collection of 22 patterns, corresponding to a total isothermal holding time of approximately 55 min. For the stress-applied experiment, the tensile stress was immediately applied at the end of the collection of the first pattern, causing a time interval of ~ 43 s between the first two measurements.

**Figure 2:**
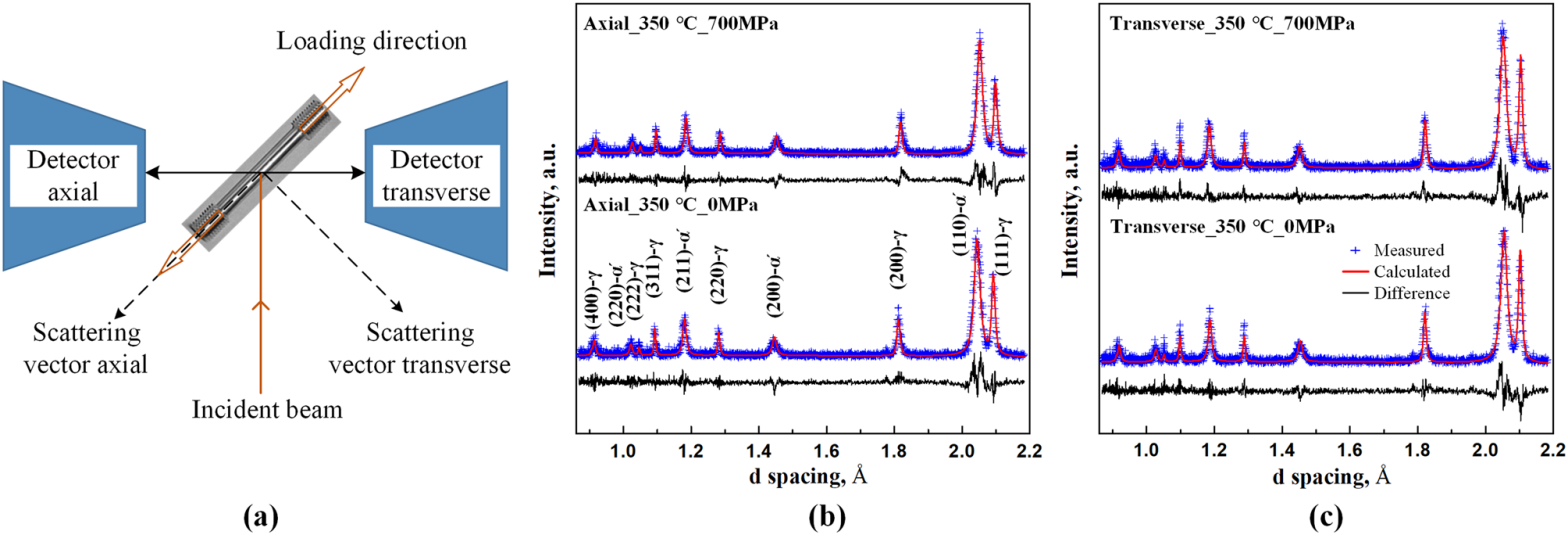
(**a**) The setup of ENGIN-X diffractometer at ISIS. Demonstration of full-profile Rietveld refinement via GSAS for two diffraction patterns from axial direction (**b**) and two diffraction patterns from transverse direction (**c**). The results in (**b**,**c**) show measured data points, calculated curves and the difference between measured and calculated curves.

Figure 2a shows the setup of the neutron diffraction measurements. The tensile direction was oriented 45° with respect to the incident beam. Two detectors mounted perpendicular to the incident beam enabled the simultaneous acquisition of the diffraction patterns from both the axial direction (prior RD) and transverse direction. In the stress-free course, only one end of the specimen was mounted to the stress rig to allow for its free volume change. Diffraction patterns were collected over a wide range of lattice spacings. The GSAS II package was used for the full-profile Rietveld refinement to determine the lattice parameter which was further used for the calculation of phase strains. In Fig. 2b,c, the Rietveld refinement is demonstrated for the patterns obtained from axial and transverse directions before and after mechanical loading at 350 °C. In the present results, no visible peak splitting indicative of any tetragonality of martensite was observed in diffraction patterns. This is consistent with the results observed for the martensite phase in a Co-free variant of the experimental steel (Fe–13Cr–0.47C) indicating that martensite loses most of its tetragonality at 300 °C^27^. Accordingly, the crystal structure of martensite can be assumed to be body-centered cubic and can be described by one lattice parameter only.

The microstructure of a specimen in the quenched and partitioned condition was characterized by optical microscopy (OM) and electron backscatter diffraction (EBSD). To reduce the risk of preparation-induced martensite formation, the specimen was mechanically ground under the flow of hot water (nearly 80 °C). This was followed by polishing with 6 μm and 3 μm diamond suspensions and a final polish with a non-crystallizing colloidal silica suspension. Etching for OM was done by using the Beraha I solution. After electropolishing, EBSD measurements with a step size of 0.35 μm were done in a Zeiss Auriga field-emission scanning electron microscope.

## Results and discussions

### Microstructure

**Figure 3:**
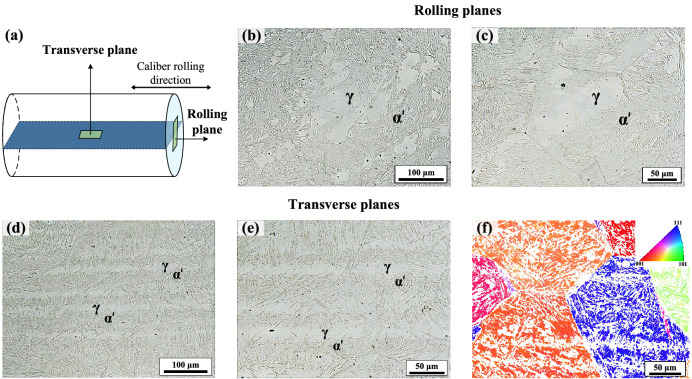
(**a**) Schematic of specimen planes as referred to in the presented micrographs; (**b**,**c**) optical micrographs of the rolling plane; (**d**,**e**) optical micrographs of the transverse plane. In (**b**–**e**) ***α′*** and ***γ*** phases provide dark and light contrasts, respectively. (**f**) EBSD inverse pole figure map of austenite in the transverse plane, showing crystal directions normal to the plane of view in color.

The schematic in Fig. 3a shows the polish planes for microstructural examinations. Figure 3b–e show optical micrographs corresponding to the rolling and transverse planes, respectively. After etching with Beraha I solution, the austenite phase is less attacked by the etchant and appears bright, while martensite phase gives a relatively dark contrast. A closer examination of the optical micrographs indicated that—in spite of the careful metallographic specimen preparation—some of the austenite regions have transformed to martensite during specimen preparation. Nevertheless, the fact that the freshly formed martensite did not contain tempering carbides still provided an adequate contrast for the identification of martensite and prior austenite. The presence of a fiber-banded microstructure is also evident in the EBSD inverse pole figure map of austenite as shown in Fig. 3f.

**Figure 4:**
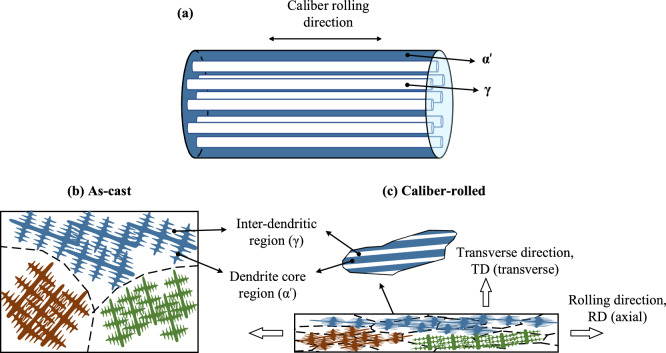
Schematic illustrations of (**a**) the idealized microstructure consisting of fibre-like austenitic regions surrounded by transformed martensite regions (cf. micrographs in Fig. 3), (**b**) the dendritic as-cast microstructure and (**c**) the formation of a fibre-banded microstructure during caliber rolling.

Based on the microstructural examinations, the microstructure can be described to consist of relatively coarse fibre-like austenitic regions surrounded by transformed martensite regions (Fig. 4a). The latter regions also contained a small fraction of interlath austenite. As the hard martensite phase forms a continuous network, it is expected to exert a large influence on the deformation of austenite when the phase mixture is subjected to an external loading.

In steels, there is a general consensus that the banded microstructure^28,29^ arises because of the microsegregation of alloying elements which can be traced back to the dendritic nature of the solidification process^30,31^. The formation of a banded structure is schematically illustrated in Fig. 4b,c. During solidification, dendrite nucleation and growth leads to the segregation of majority of alloying elements to the remaining liquid, namely inter-dendritic regions once the solidification is complete. In particular, the segregation pattern is controlled by the solidification mode (ferritic vs austenitic), cooling condition, and the solubility of alloying elements in the coexisting solid and liquid phases^32^. In Fig. 4b, several dendrites aligned parallel to one another are shown within a few grains in the as-cast condition. The dendrite cores and arms are expected to have a relatively lower alloy content as the alloying elements have segregated to inter-dendritic regions during the solidification process. Upon the subsequent hot-rolling, the segregation pattern of alloying elements is elongated in the rolling direction (Fig. 4c). Hot rolling is also accompanied by grain refinement due to dynamic/static recrystallization. As a result, the elongated dendrites may extend across many grain boundaries after hot rolling. At RT, the dendritic regions with a low solute content and inter-dendritic regions with a high solute content appear in the form of ***α′*** and ***γ*** phases, respectively. The microstructural banding is retained during the subsequent Q&P treatment.

### Stress-free and stress-applied measurements

**Figure 5:**
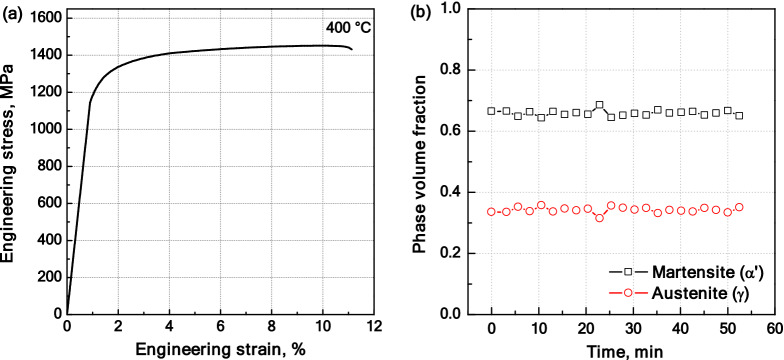
(**a**) Engineering stress–strain curve for the quenched steel tested at 400 °C; (**b**) phase fraction evolution during stress-applied measurements.

To restrict plastic deformation during measurements under external tensile stress, the applied stress level was set to be well below the 0.2% proof stress. Figure 5a shows the engineering stress–strain curve of the quenched steel tested at 400 °C. The 0.2% proof stress was found to be 1,228 MPa. As the yield strength generally decreases at higher temperature due to thermally activated processes^33^, it is reasonable to assume that the 0.2% proof stress exceeds 1,200 MPa also at 350 °C. Therefore, the applied stress was set to 700 MPa. Phase fraction evolutions of *α′* and *γ* phases based on stress-applied diffraction measurements are shown in Fig. 5b. The average martensite fraction (f_**α′**_) is approximately 66 vol% and the steady temporal evolution of the phase fraction confirms the absence of phase transformation during stress-applied measurements.

**Figure 6:**
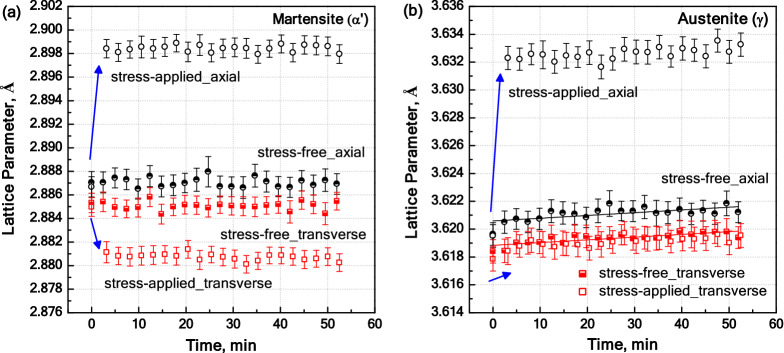
Lattice parameter evolutions for (**a**) ***α′*** and (**b**) ***γ*** phases during stress-free and stress-applied experiments. Arrows indicate changes in **a**_**α′**_ and **a**_**γ**_ due to the stress application.

Figure 6 shows the lattice parameter evolutions of austenite (**a**_**γ**_) and martensite (**a**_**α′**_) for both axial and transverse directions. The average error associated with the Rietveld refinement procedure is estimated to be ± 0.0008 Å. In the course of stress-free measurements, as shown in Fig. 6a, **a**_**α′**_ remains nearly constant indicating that the tempering reactions associated with a change in the solute C content of martensite have occurred at lower temperatures during heating. For the austenite, on the other hand, the results in Fig. 6b show a slight increase in **a**_**γ**_ along both axial and transverse directions. The increase in **a**_**γ**_ can be justified by the C enrichment of austenite during stress-free measurements. The C enrichment of austenite without noticeable changes in the solute C content of martensite implies that the C atoms migrated into the austenite have been supplied by those segregated at lattice distortions in martensite and phase boundaries rather than the solute C in martensite^27^. The segregation of C to defects in the martensite can happen even at RT according to atom probe tomography investigations^7^. The C enrichment of austenite occurs mostly within the first 2 to 3 measurements corresponding to a time of ~ 5 min and becomes sluggish during the rest of the steps. Similar observations have been made during the high-temperature (HT) XRD measurements using a Co-free variant of the present steel^12^. In the latter case, majority of the C partitioning had already occurred during heating to 350 °C. Therefore, the fraction of C that continues to enrich in the austenite during the current neutron diffraction measurements is only a small fraction of the C already partitioned into the austenite before the onset of the measurements. This facilitated the intended analysis of the stress–strain partitioning. In the course of the stress-applied experiment, **a**_**α′**_ remains constant along both axial and transverse directions, while **a**_**γ**_ grows at a rate comparable to the stress-free experiment. The comparable evolution of **a**_**γ**_ with and without stress application confirms that the application of stress does not enhance the extent of C partitioning. Therefore, the lattice parameters based on stress-free measurements were used to estimate the relatively small effect of C enrichment on the measured **a**_**γ**_ values during the measurements.

According to the Fick’s second law, the diffusion distance is proportional to square root of time ($\sqrt{t}$)^34^. Assuming that the increase in the lattice parameter of austenite arising from C partitioning changes proportional to the diffusion distance, the increase in the lattice parameter attributable to the C enrichment is also expected to be proportional to $\sqrt{t}$. Our efforts to plot a reasonable square root fit to the lattice parameters of austenite in stress-free measurements (Fig. 6b) was not successful. This can be explained by the steady decrease in the C (in martensite) that is available for partitioning. Therefore, the evolution of **Δa**_γ_ with time for the case of stress-free condition was approximated by means of linear fitting to the **a**_γ_ values of stress-free measurements (dashed lines in Fig. 6b).

Quantitative analysis of the change in the solute C content of austenite (∆C_γ_) was made using the following equation:

1 $$\Delta {\text{C}}_{\gamma } ({\text{wt}}\% ){\text{ = }}\frac{{\Delta {\text{a}}_{\gamma } }}{{\beta _{\gamma } }}(\Delta {\mathbf{a}}_{\gamma } \;{\text{in}}\;[{\AA}])$$

where *β*_γ_ is a constant and can take values between 0.033 and 0.047^35–38^. Since recent XRD measurements^39^ yielded a value almost exactly matching the value 0.044 reported in^36^, the latter value was used for the estimation of the solute C content in austenite.

The slope of the fitted line in axial direction is comparable to that in transverse direction. This implies that the lattice distortion caused by the time-dependent C enrichment of austenite causes a nearly isotropic expansion of the lattice parameter, which is consistent with the regularity of octahedral sites in austenite^40^. At the end of the stress-free experiment, the C enrichment of austenite, ∆C_γ_, based on the average of the two linear fits is about 0.0233 ± 0.018 wt % (~ 5.07 ± 0.04% of the nominal C concentration of the steel). This estimation is in agreement with our previous results in which changes in the lattice parameter of austenite due to its C enrichment were measured by HT XRD measurements^12^.

Referring to Fig. 6b, the temporal evolution of **a**_**γ**_ along transverse and axial directions is comparable to those for the stress-free condition. The comparable lattice expansion of austenite after the application of stress shows that there is no influence of external stress on C partitioning kinetics. Furthermore, it appears unlikely that the application of stress has resulted in an abrupt instantaneous boost in the C concentration of austenite. With this assumption, the stress and strain partitioning upon the application of stress was studied by subtracting the lattice parameter increment caused by the C enrichment of austenite. The stress-free measurement data provided the reference values for the latter subtraction procedure.

### Stress and strain partitioning

By subtracting the lattice parameter under stress-free condition (at 0 MPa) from stress-applied condition (at 700 MPa) (${a}_{p}={a}_{p}^{700 MPa}-{a}_{p}^{0 MPa}$, *p* = ***α′*** or ***γ***), the phase strain (${\varepsilon }^{p}$) can be calculated based on the following equation:

2 $${\varepsilon }^{p}=\frac{{a}_{p}-{a}_{p, 0}}{{a}_{p, 0}}$$

where ${a}_{p, 0}$ is the lattice parameter obtained from the first diffraction pattern (of the stress-applied scheme), namely before the application of stress at 350 °C.

**Figure 7:**
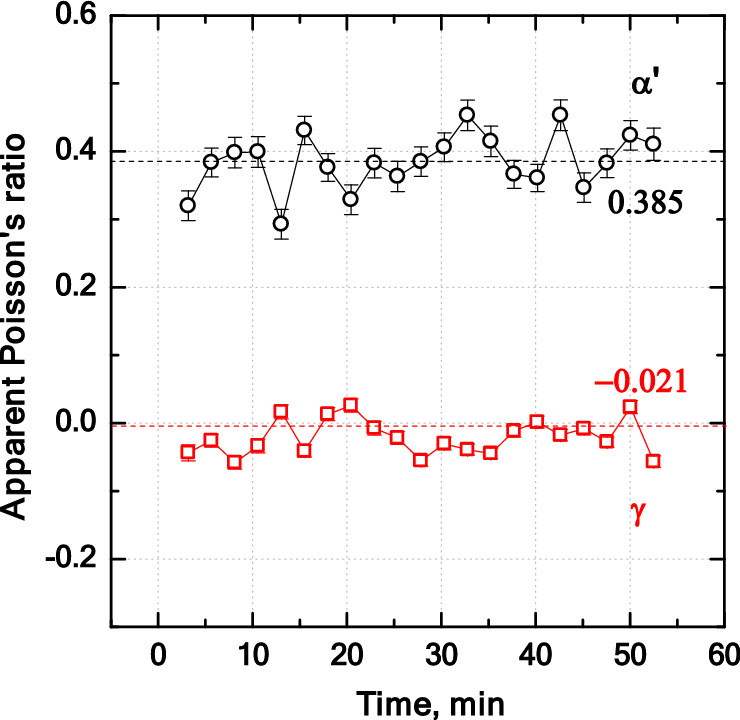
Apparent Poisson’s ratio of phases ***α′*** and ***γ*** during stress-applied measurements. The values were calculated using − ${\varepsilon }_{transverse}/{\varepsilon }_{axial}$.

For an isotropic specimen (e.g. polycrystals) subjected to uniaxial tensile stress, the Poisson's ratio is a measure of the contraction in directions perpendicular to the direction of straining (transverse direction) relative to that in the axial direction (−${\varepsilon }_{transverse}/{\varepsilon }_{axial}$). When used within their design limits (below yield point), most steels and rigid polymers exhibit values of the order of 0.3^41^. Apparent Poisson’s ratios ($v$) of ***α′*** and ***γ*** phases during measurements under stress were calculated as (− ${\varepsilon }_{transverse}/{\varepsilon }_{axial}$) and are given in Fig. 7. The average apparent Poisson’s ratios for ***α′*** and ***γ*** phases are 0.385 and − 0.021, respectively. The abnormal near-zero elastic strains in the austenite phase in directions perpendicular to the tensile loading direction, as can be also inferred from Fig. 6b, is expected to arise due to the development of a stress state much different from that expected upon loading of a fully austenitic specimen^42^. This peculiar observation is explained by the interactions of austenite with the coexisting martensite as discussed in the following.

The relation between stress and strain in multiphase alloys is usually described by the law of mixtures^43,44^ through defining a mixture in which either stress or strain in all phases are equal^45^. Given the alignment of the microstructure bands of ***α′*** and ***γ*** parallel to the tensile loading axis in the present study, it can be assumed that along the axial direction, ***α′*** and ***γ*** phases experience an equal strain (${\varepsilon }_{axial}^{\gamma }={\varepsilon }_{axial}^{{\alpha }^{^{\prime}}}$). The equal strain assumption is also commonly used to describe the behavior of fiber-reinforced composite materials loaded parallel to the length of fibers^46^.

As mentioned previously, the applied tensile stress of 700 MPa is far below the 0.2% proof stress of the steel (as shown in Fig. 5a). Due to the higher yield strength of ***α′*** compared to that of the ***γ***, the deformation of ***α′*** under the stress-applied condition in the present work can be assumed to be entirely elastic. Once the strain tensor components along axial and transverse directions in martensite (${\varepsilon }_{axial}^{{\alpha }^{^{\prime}}}$ and ${\varepsilon }_{transverse}^{{\alpha }^{^{\prime}}}$) are obtained via experimental measurements, the stress tensor components (${\sigma }_{axial}^{{\alpha }^{^{\prime}}}$ and ${\sigma }_{transverse}^{{\alpha }^{^{\prime}}}$) can be determined by using the Hooke’s law. The Hooke’s law for the Cartesian coordinates system with a set of x, y, and z orthogonal axes can be written in the following form^47^:

3 $${\sigma }_{x}=\frac{E}{\left(1+\nu \right)\left(1-2\nu \right)}\left[\left(1-\nu \right){\varepsilon }_{x}+\nu \left({\varepsilon }_{y}+{\varepsilon }_{z}\right)\right]$$

4 $${\sigma }_{y}=\frac{E}{(1+\nu )(1-2\nu )}[\left(1-\nu \right){\varepsilon }_{y}+\nu ({\varepsilon }_{z}+{\varepsilon }_{x})]$$

5 $${\sigma }_{z}=\frac{E}{(1+\nu )(1-2\nu )}[\left(1-\nu \right){\varepsilon }_{z}+\nu ({\varepsilon }_{x}+{\varepsilon }_{y})]$$

where E is the Young’s modulus and $v$ is the Poisson’s ratio. In the present case, the axial direction of the tensile specimens can be taken as the x direction and two orthogonal directions normal to the tensile loading direction as y and z direction. The fibre-banded microstructure in the present case imposes that the stress in all directions perpendicular to the axial direction ${\sigma }_{transverse}^{{\alpha }^{^{\prime}}}$ should be equal. In other words, y and z directions in Eqs. (3)–(5) may be treated as the same and may be treated as the transverse direction in the present case. Therefore, the Hooke’s law equations can be adapted for the present case and rewritten as:

6 $${\sigma }_{axial}^{{\alpha }^{^{\prime}}}=\frac{{E}^{{\alpha }^{^{\prime}}}}{\left(1+{\nu }^{{\alpha }^{^{\prime}}}\right)\left(1-2{\nu }^{{\alpha }^{^{\prime}}}\right)}\left[\left(1-{\nu }^{{\alpha }^{^{\prime}}}\right){\varepsilon }_{axial}^{{\alpha }^{^{\prime}}}+2{\nu }^{{\alpha }^{^{\prime}}}{\varepsilon }_{transverse}^{{\alpha }^{^{\prime}}}\right]$$

7 $${\sigma }_{transverse}^{{\alpha }^{^{\prime}}}=\frac{{E}^{{\alpha }^{^{\prime}}}}{\left(1+{\nu }^{{\alpha }^{^{\prime}}}\right)\left(1-2{\nu }^{{\alpha }^{^{\prime}}}\right)}\left[{\nu }^{{\alpha }^{^{\prime}}}{\varepsilon }_{axial}^{{\alpha }^{^{\prime}}}+{\varepsilon }_{transverse}^{{\alpha }^{^{\prime}}}\right]$$

Since the elastic constants of martensite are similar to ferrite, we used the parameters of ferrite instead. All the parameters used in the current calculations are listed in Table 2. In martensitic-austenitic stainless steels, the martensite phase acts as the harder phase dominating the deformation of both phases. Along the transverse direction, due to the requirement of force balance between ***α′*** and ***γ*** phases at phase boundaries, the stress in austenite depends on the volume fraction ratios according to the following equation:

8 $${\sigma}_{transverse}^{\gamma }={-\frac{{f}^{{\alpha }{^{\prime}}}}{{f}^{\gamma}}{\sigma}_{transverse}^{{\alpha}^{\prime}}}$$

where *f* represents the volume fraction of the superscripted phase and the negative sign reflects the fact that the stresses in ***α′*** and ***γ*** phases are of opposite sign.

**Table 2:** High temperature (T) Young’s modulus (*E*) and Poisson’s ratio ($v$) of ***α′*** and ***γ*** phases^48^.

T, °C	***γ***		***α′***	
	*E*, GPa	$v$	*E*, GPa	$v$
350	175	0.313	200	0.287

Given the elastic nature of the microstrains measured by neutron diffraction, the Hooke’s law can be used for the calculation of the elastic stresses in ***γ*** in the axial direction:

9 $${\sigma }_{axial}^{\gamma }={\varepsilon }_{axial}^{\gamma }\cdot {E}^{\gamma }+2{\nu }^{\gamma }{\sigma }_{transverse}^{\gamma }$$

Once the stress state of ***γ*** in the axial direction is known, the elastic strain caused by its stress state can be calculated as follows:

10 $${\varepsilon }_{transverse, calc.}^{\gamma }=\frac{1}{{E}^{\gamma }}\left[\left(1-{\nu }^{\gamma }\right){\sigma }_{transverse}^{\gamma }-{\nu }^{\gamma }{\sigma }_{axial}^{\gamma })\right]$$

**Figure 8:**
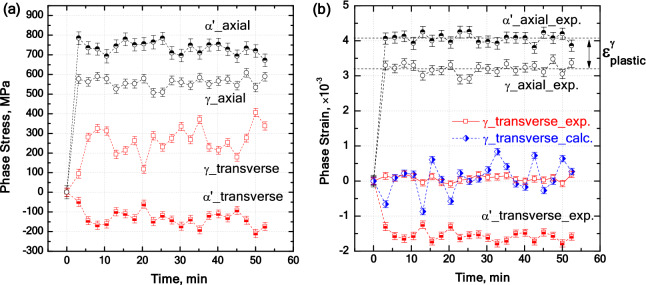
(**a**) Stress partitioning and (**b**) strain partitioning in axial and transverse direction for the indicated phases under an external tensile stress of 700 MPa; (**b**) also shows the calculated and experimental strains in austenite along the transverse direction.

The calculated stresses and strains in axial and transverse directions of ***α′*** and ***γ*** are plotted in Fig. 8a. For stress calculations, possible preexisting residual stresses in phases prior to neutron diffraction measurements, e.g. thermally-induced stresses, were neglected. Along the external tensile loading axis, stress in ***α′*** is nearly 730 MPa and exceeds that in ***γ*** at about 560 MPa. The latter value significantly surpasses the typical yield strength during uniaxial tensile testing of austenitic steels^49^. Provided that the used assumption of equal strain in ***α′*** and ***γ*** phases along the axial direction holds perfectly, the difference between the strains in ***α′*** and ***γ*** phases (Fig. 8b) can be used to estimate the actual plastic strain experienced by the *γ* phase in the axial direction. Such a calculation returns a plastic strain of only ${\varepsilon }_{plastic}^{\gamma }=$ 0.087% in the austenite phase. Accordingly, no peak broadening effect was detected in the diffraction patterns and no visible plasticity caused by microyielding could be inferred from the stress–strain curve (Fig. 5a).

The small level of plastic strain in ***γ*** appears at first glance not to be compatible with the high stress level of 560 MPa carried by the austenite phase in the axial direction. This observation can be explained by the development of a multiaxial stress state in austenite, as outlined in the following, resulting in a reduction in the deviatoric component of stress. The abnormal positive strain in the transverse direction of ***γ*** phase implies the existence of tensile forces that oppose its lateral contraction. The opposing forces are caused by the predominant and stronger ***α′*** phase as it responds to the applied axial stress by contracting in the transverse direction. The lateral contraction of ***α′*** phase in turn induces a tensile stress of approximately 260 MPa in the ***γ*** phase, opposing its transverse contraction. Accordingly, the equivalent von Mises stress is calculated to be approximately 300 MPa, which is comparable to the elastic limit of an austenitic stainless steels with a similar chemical composition at 200 °C^49^. Considering that the austenite is subjected to a multiaxial stress state^50,51^, as imposed by the coexisting martensite phase, a large hydrostatic component of the stress tensor might have delayed yielding^52^.

It is known that dissimilar CTE of austenite (***γ***) and ferrite/martensite (***α***/***α′*** )^17,18,24^ in banded microstructures could induce microstresses^10,23^. In the present case, such stresses can develop during processing steps where ***γ*** and ***α′*** coexist. These are, on the one hand, temperatures below the martensite start temperature of the steel (~ 138 °C,^53^) as the steel is oil cooled from the solution annealing temperature and, on the other hand, during heating from RT to 350 °C for neutron diffraction measurements. Cooling will have an additional contribution due to the expansion associated with martensitic transformation. Further factors that might aid the residual stress development are the length changes associated with tempering reactions in ***α′*** and carbon partitioning between ***α′*** and ***γ*** during heating to 350 °C for neutron diffraction measurements.

Given the higher CTE of ***γ*** than ***α′*** in the banded structure, oil quenching will induce tensile stresses in ***γ*** parallel to the bands. Heating of a ***γ*** + ***α′*** banded microstructure, on the other hand, will cause compressive microstresses in ***γ*** parallel to the bands. Therefore, heating to 350 °C will initially cancel out any residual stresses that might have developed during oil quenching and then causes a reversal of the sign of stress. At the beginning of neutron diffraction measurements at 350 °C, residual compressive microstresses are likely to exist in ***γ*** parallel to the bands, and tensile stresses in the transverse direction. For ***α′*** , microstresses will be of an opposite sign. Preexisting compressive stresses in the ***γ*** phase parallel to the bands are another possible reason contributing to the negligible yielding of austenite during external tensile loading to 700 MPa.

**Figure 9:**
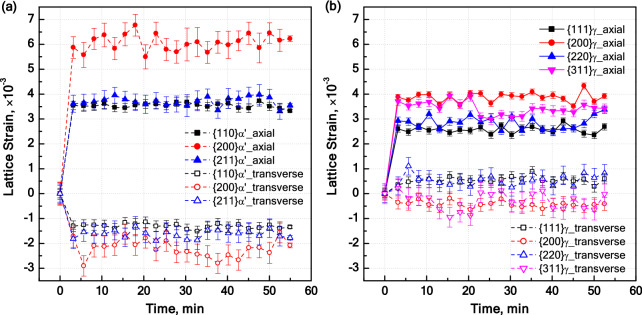
Evolution of lattice strains for different reflections of (**a**) martensite and (**b**) austenite. Lattice strains are shown for both axial and transverse directions.

The elastic strains for different lattice planes are summarized in Fig. 9 and indicate a conyiderable anisotropy of elastic strains. The relative magnitudes of elastic strains in ***α′*** phase (Fig. 9a) in the loading direction are in an almost perfect agreement with the reported stiffness values for ferritic steels^52^. In other words, elastic strains are inversely proportional to the elastic moduli, namely E_200,α′_ < E_211,α′_ < E_110,α′_. The relative magnitude of elastic strains for ***γ*** phase (Fig. 9b) in the loading direction are in reasonable agreement with the reported elastic moduli, namely E_200,γ_ < E_311,γ_ < E_220,γ_ < E_111,γ_^24^. If peak shifts due to the lattice faults in ***γ*** phase are neglected, the small discrepancy between the relative elastic moduli and lattice strains can be attributed to the extent to which the used stiffness values for ***γ*** phase are applicable to the present steel.

## Conclusions

The influence of segregation-induced microstructure bands of ***α′*** and ***γ*** phases aligned by caliber rolling on the partitioning of stress and strain between the phases was investigated. For this purpose, tensile specimens of a quenched and partitioned Co-added stainless steel were tensile loaded parallel to the direction of the bands and the lattice parameters in the axial and transverse directions were simultaneously measured by time-of-flight neutron diffraction experiments at 350 °C. Monitoring the temporal evolution of lattice parameters under stress-free and stress-applied holding indicated that stress application had no evident effect on boosting the C enrichment of ***γ*** phase. The observation of a negative apparent Poisson’s ratio of − 0.021 for the ***γ*** phase upon the application of a tensile stress of 700 MPa indicated a constraint on the free transverse straining of ***γ*** phase. The constraint was found to arise from the fibre-banded microstructure. In simple terms, the lateral contraction of the ***α′*** phase imposed an interphase tensile stress in the transverse direction of the ***γ*** phase, thereby opposing its contraction. The induced tensile stress of approximately 260 MPa in the ***γ*** phase opposed its transverse contraction in response to its actually positive Poisson’s ratio. This observation was explained by a basic consideration of the elasticity theory. The multiaxial stress state developed in the ***γ*** phase resulted only in a negligible plastic deformation of ***γ*** phase and a large deviation from the level of plastic strain expected if single phase ***γ*** were uniaxially loaded. The analysis of stress and strain partitioning with the present Q&P steel can be used to interpret the observations made for further two-phase alloys with banded microstructures.
