# Author Correction: Neutron diffraction analysis of stress and strain partitioning in a two-phase microstructure with parallel-aligned phases

**DOI:** 10.1038/s41598-020-74028-6

**Published:** 2020-10-07

**Authors:** Qiuliang Huang, Ran Shi, Ondrej Muránsky, Hossein Beladi, Saurabh Kabra, Christian Schimpf, Olena Volkova, Horst Biermann, Javad Mola

**Affiliations:** 1 Institute of Iron and Steel Technology, Technische Universität Bergakademie Freiberg, Leipziger Str. 34, 09599 Freiberg, Germany; 2 Material Design and Structural Integrity Lab, Faculty of Engineering and Computer Sciences, Osnabrück University of Applied Sciences, Albrecht St. 30, 49076 Osnabrück, Germany; 3 Australian Nuclear Science and Technology Organisation (ANSTO), Sydney, NSW 2234 Australia; 4 Institute for Frontier Materials, Deakin University, Geelong, VIC 3216 Australia; 5 ISIS Neutron and Muon Facility, The Rutherford Appleton Laboratory, Oxfordshire, UK; 6 Institute of Materials Science, Technische Universität Bergakademie Freiberg, Gustav-Zeuner-Straße 5, 09599 Freiberg, Germany; 7 Institute of Materials Engineering, Technische Universität Bergakademie Freiberg, Gustav-Zeuner-Straße 5, 09599 Freiberg, Germany; 8 Present Address: Institute of Energy Process Engineering and Chemical Engineering, Fuchsmühlenweg 9, 09599 Freiberg, Germany

Correction to: *Scientific Reports* 10.1038/s41598-020-70299-1, published online 11 August 2020

The original version of this Article contained an error in Affiliation 5, which was incorrectly given as ‘Spallation Neutron Source, The Rutherford Appleton Laboratory, Oxfordshire, UK’. The correct affiliation is listed below:

ISIS Neutron and Muon Facility, The Rutherford Appleton Laboratory, Oxfordshire, UK.

This error has now been corrected in the HTML and PDF versions of the Article.

